# Applications of Selected Nanoencapsulated Indigenous Essential Oils in Medicine, Food, and Agriculture: A Review

**DOI:** 10.3390/foods15111942

**Published:** 2026-06-01

**Authors:** Ongeziwe Sinazo Wutu, Babalwa Mpambani, Clarissa Marcelle Naidoo

**Affiliations:** 1 Dohne Agricultural Development Institute, Private Bag X 15, Stutterheim 4930, South Africa; babalwa.mpambani@ecagriculture.gov.za; 2 Department of Biology and Environmental Sciences, School of Science and Technology, Sefako Makgatho Health Science University, Pretoria 0204, South Africa; clarissa.naidoo@smu.ac.za

**Keywords:** applications, indigenous essential oils, nanoencapsulation, nanotechnology

## Abstract

The growing demand for natural, safe, and sustainable bioactive compounds has sparked interest in indigenous essential oils (EOs) for their antimicrobial, antioxidant, and therapeutic properties. Their practical applications are often limited by poor solubility, volatility, and susceptibility to degradation when exposed to light, heat, and or oxygen. The literature lacks exploration of the indigenous EOs in nanoencapsulation studies. Using nanosystems and carriers, the oil can be delivered to targeted areas over a longer period. This is useful for various applications, including biopesticides, regenerative medicine, gene therapy, textiles, and antimicrobial coatings. Studies reveal that nanoencapsulated EOs exhibit higher insecticidal and antimicrobial activity than free oil. In this review, we observed that *Lippia javanica* is the most used EO in nanoencapsulation processes. This may be attributed to its broad spectrum of biological activities and its wide distribution in South Africa. This review examines the applications of selected nanoencapsulated indigenous EOs of the Eastern Cape province in medicine, food, and agriculture. The findings underscore the potential of nanoencapsulation to transform indigenous EOs into multifunctional agents that can support health, food security, and sustainable agricultural practices, while calling for further research on safety, regulatory frameworks, and commercialization pathways.

## 1. Introduction

Essential oils are heterogeneous mixtures of low-molecular-weight volatile compounds biosynthesized by aromatic plants, where they function in ecological roles such as chemical signalling, allelopathy, and defence against pathogens [1]. Their extensive historical use in traditional medicine, agriculture, and industrial applications is attributed to their broad-spectrum bioactivity [2,3]. Pharmacological studies have demonstrated that essential oils exhibit antimicrobial, antiviral, anti-inflammatory, antioxidant, and spasmolytic properties, mediated by their diverse phytochemical constituents [4,5].

These oils contain a diverse range of bioactive compounds, including terpenes, phenolics, and aldehydes, which are responsible for their broad spectrum of biological activities. The interest in essential oils has surged in recent decades, owing to the growing preference for natural products over synthetic alternatives, especially in fields such as medicine, where their higher molar weight and better molecular rigidity have made them useful in chronic diseases [6,7]. Questions have been raised regarding the long-term health effects of synthetic food preservatives such as sodium benzoate, butylated hydroxyanisole (BHA), and sulphides. There have been arguements that children who eat junk food are more prone to hyperactivity and inattentive behaviour, which can lead to academic problems later in life [8]. Researchers [9,10] agree with these claims, adding that people who consume food products containing traces of these preservatives are more prone to heart problems. In agriculture, synthetic fertilizers are known to decrease soil organic content and contribute to the depletion of soil mineral nutrients. They also harden the soil, which can reduce its fertility and pose environmental hazards. In addition to altering the soil’s pH level, these fertilizers can also increase the resistance of pests and have an adverse effect on the soil’s crust. Moreover, they are prone to leaching, which leads to the contamination of water and therefore has a negative effect on aquatic animals [11,12]. On the other hand, EOs can help improve the quality of the soil by enhancing microbial activity and texture. Additionally, EOs are environmentally friendly, therefore preventing water contamination [13,14].

From a market perspective, EOs can offer a seasonal supply, as well as export potential. For these reasons, they are one of the protected commodities and remain a high priority for the South African government [15]. A case study was conducted by Essential Amathole, an agricultural enterprise that aims to reduce poverty in rural communities through the cultivation of essential oils. The survey, undertaken in the Amathole district in the Eastern Cape province, revealed that the use of EOs could help address the socio-economic conditions of the communities. The quality of the extracted oils from the trials was above average, demonstrating the potential to establish an enterprise hub for EO production. In addition, the number of employees working for the company increased significantly during the five-year period following its startup, indicating the opportunities that the EO industry possesses [16,17]. Among the companies that focus on the research and distribution of specialty agricultural products is ICA International Chemicals. It specializes in the use of essential oils in the production of biopesticides.

With all these advantages for EOs, their practical use is unfortunately often limited by poor solubility, volatility, and susceptibility to degradation when exposed to light, heat, or oxygen. For example, due to the hydrophobic nature of essential oils, it has been challenging to incorporate them into low-fat food products [18]. Their intense flavour and aroma also limit their application in certain food products. Although this issue is considered a challenging problem, it can be solved through encapsulation [19,20]. These challenges reduce their effectiveness and shelf life in various applications. This is where innovations such as nanotechnology come into play. Nanotechnology emerged as scientists became more inquisitive about the behaviour of materials and matter at the atomic and molecular levels [21]. At scales smaller than 100 nanometres (a nanometer is one-billionth of a meter), materials exhibit unique properties, such as changes in electrical conductivity, reactivity, and strength, that are not observed in bulk materials. Understanding these properties opened up new possibilities for applications that traditional science and engineering could not achieve [22]. Thus, the basic principle of nanoencapsulation is the process of enclosing or trapping bioactive molecules (like essential oils or drugs) within nanoparticles (NPs) or nanocarriers [23,24]. Examples include various types of lipid- or polymeric nanoparticle-based products, such as nanocapsules, liposomes, dendrimers, nanogels, and nanosuspensions, which are used to encapsulate and release substances [25,26]. The result is a system where the active substance is protected by a nanostructured shell or matrix, which can control the release and improve its delivery [27,28,29,30,31]. Nanoencapsulation is a promising technique that addresses these limitations by encapsulating essential oils in nanoscale carriers, such as liposomes, solid lipid NPs, and nanostructured lipid carriers [32]. This technology enhances the stability, controlled release, and bioavailability of EOs, enabling more effective delivery and a wider range of applications.

In the medical field, nanoencapsulated EOs are gaining attention for their potential use in drug delivery, wound healing, and the treatment of various diseases due to their anti-inflammatory, antimicrobial, and antioxidant properties. Their encapsulation not only enhances their therapeutic efficacy but also minimizes adverse side effects by ensuring targeted and sustained release [33,34]. In the food industry, the nanoencapsulation of essential oils provides an innovative approach to preserving food quality, preventing spoilage, and extending shelf life [35,36]. Indigenous EOs, such as those derived from *Pelargonium graveolens* (rose geranium) and *Lippia javanica* (lemon bush), have natural antimicrobial properties, making them ideal candidates for inclusion in food packaging materials or as additives in functional foods. Nanoencapsulation also helps preserve the volatile compounds of essential oils, maintaining their aroma and flavour while enhancing safety and stability. Agricultural applications of nanoencapsulated essential oils focus on pest control, plant growth regulation, and disease management. The controlled release of these oils, such as those from *Artemisia afra* (umhlonyane) and *Helichrysum odoratissimum* (impepho), can provide effective, eco-friendly alternatives to chemical pesticides, promoting sustainable farming practices. Additionally, their antimicrobial properties help to reduce post-harvest losses and protect crops from various pathogens [37,38].

This review aims to explore the potential applications of selected nanoencapsulated indigenous EOs of *L. javanica*, *A. afra, Agathosma betulina, Leonotis Leonurus, H. odoratisimum,* and *P. graveolens*, with the exception of *Cymbopogon citratus*. Although *C. citratus* is not native to the region, its extensive cultivation and research on nanoencapsulation make it an ideal candidate for analytical and application-focused perspectives. The EOs were explored based on their applications in three key sectors—medicine, food, and agriculture—focusing on their efficacy, mechanisms of action, challenges, and future potential.

Due to limited research information on *Cymbopogon validus*, which is indigenous to the Eastern Cape province, the focus will be on *C. citratus*, which is native to Africa and cultivated in the Eastern Cape province. Through this exploration, the study seeks to provide a deeper understanding of how nanoencapsulation can revolutionize the use of indigenous essential oils found in the Eastern Cape province of South Africa, offering sustainable solutions to pressing global challenges in health, food security, and environmental sustainability.

## 2. Materials and Methods

**Figure 1:**
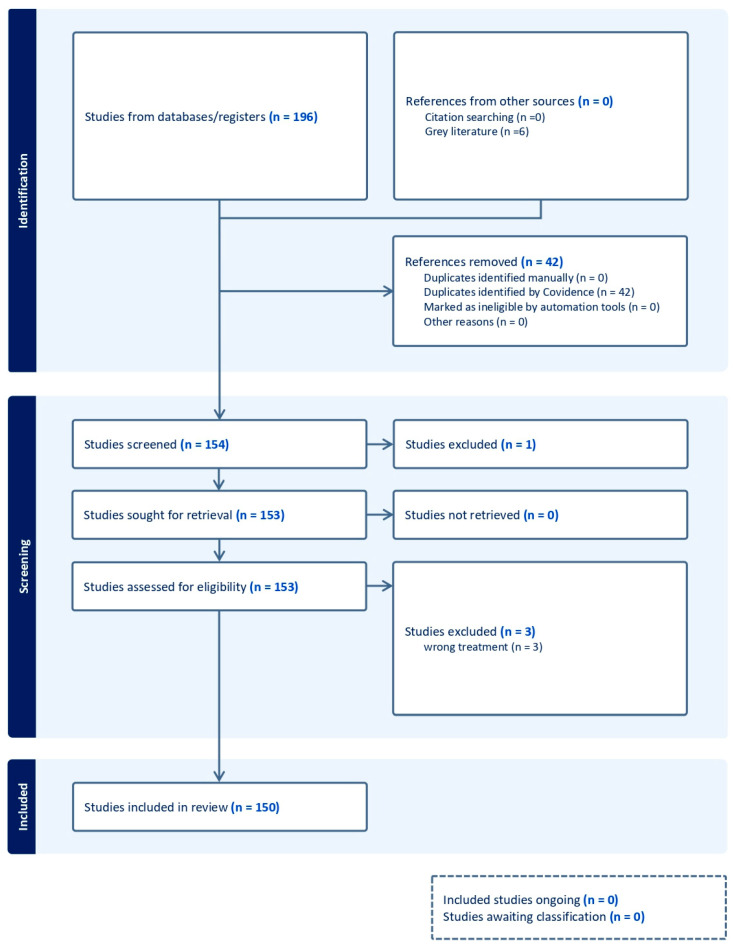
The present manuscript was developed using the PRISMA methodology, which is illustrated in a flow chart.

This study adopted a critical narrative review approach to evaluate and synthesize the existing literature on the applications of selected nanoencapsulated indigenous essential oils in medicine, food systems, and agriculture [39]. A PRISMA diagram (Figure 1) has been provided using Covidence systematic review software [40] (https://www.covidence.org/ accessed on 30 March 2026). This approach emphasizes critical interpretation, conceptual integration, and the identification of knowledge gaps rather than exhaustive quantitative synthesis. The objective was to assess technological advancements, functional efficacy, and translational potential across sectors.

### 2.1. Literature Search Strategy

A structured literature search was conducted using major scientific databases, including Scopus, Web of Science, PubMed, ScienceDirect, and Google Scholar [40]. Publications from 2005 to 2026 were considered to capture recent developments in nano-delivery systems and essential oil applications. Search terms included combinations of indigenous essential oils, nanoencapsulation, nanoemulsion, liposomes, polymeric nanoparticles, solid lipid nanoparticles, food preservation, antimicrobial, antioxidant, active packaging, and biopesticide. Only peer-reviewed journal articles published in English were included. Additional relevant sources were identified through cross-referencing of bibliographies.

### 2.2. Selection Criteria

Studies were selected based on their relevance to the nanoencapsulation of plant-derived essential oils; experimental characterization of nanoformulations; demonstrated applications in medicine, food preservation, or agriculture; and reported improvements in stability, bioavailability, controlled release, or biological activity. Non-peer-reviewed materials, studies lacking nanoformulation components, and articles without sufficient methodological detail were excluded.

### 2.3. Data Extraction and Critical Analysis

Relevant data extracted from selected studies included plant species, nanoencapsulation method, physicochemical characterization (e.g., particle size, encapsulation efficiency), bioactivity outcomes, and sector-specific applications. The literature was critically analyzed to compare nano-delivery systems in terms of performance and limitations, evaluate the robustness of experimental designs, identify inconsistencies and methodological gaps, and assess scalability, regulatory considerations, and commercialization challenges. Findings were synthesized thematically under three principal domains: medical, food, and agricultural applications.

### 2.4. Limitations

As a review, this study does not provide a quantitative meta-analysis and may be subject to selection bias. However, emphasis was placed on including high-quality and recent literature to ensure balanced and evidence-based discussion.

## 3. Indigenous Essential Oils of South Africa

Indigenous EOs are volatile, aromatic phytochemical extracts derived from endemic plant species within a defined biogeographical region; in this case, South Africa [38]. They represent concentrated hydrophobic liquids comprising complex mixtures of volatile aromatic compounds, notably phenylpropanoids, monoterpenes, and sesquiterpenes. These constituents are primarily responsible for the diverse bioactive properties associated with EOs and can be extracted from various plant organs, including foliar structures [41].

Extraction techniques can be broadly classified into advanced and classical. The former methods include steam distillation, hydro-distillation, cold-pressing, and Soxhlet extraction. Although classical techniques are generally more cost-effective, they tend to produce lower yields than advanced ones [42,43,44]. Solvent extraction, cold pressing, and steam distillation are the commonly used methods of extraction, as they can be utilized for a wide range of plant materials [45,46]. Other new methods, such as microwave-assisted, supercritical CO_2_, and ultrasound extraction, are discussed in Table 1. These provide enhanced processing speeds, efficiency, and selectivity for various bioactive compounds. Supercritical CO_2_ extraction has been widely used in the production of solvent-free extracts, though its industrial potential is limited due to its high operational and capital expenses.

Studies conducted in South Africa revealed variations in the extraction methods of essential oils. In 2022, for instance, a study revealed that solvent-free microwave extraction yielded a higher quality oil of 0.29% than hydro distillation, which had a yield of 0.09% [47].

In 2024, another study revealed that the chemical constituents (terpenes, alcohols, and ketones, to name a few) of the essential oil that can be targeted or recovered by either steam distillation or hydro-distillation are the deciding factors when choosing the method [48]. Thus, there will be a loss of volatile compounds when a plant sample is exposed to higher boiling temperatures and longer extraction times. The different extraction methods used to obtain essential oils from different plant parts are summarized in Table 1.

**Figure 2:**
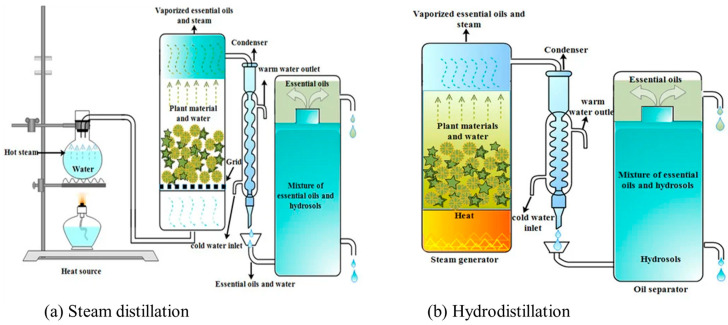
An illustration of different extraction methods of essential oils [49].

Moreover, due to plant materials being submerged in hot water for a period of time, hydrolytic reactions are common in hydrodistillation methods. Some of the oxygenated components, like phenols, tend to dissolve in the water and end up in the wastewater. This is illustrated in Figure 2.

The majority of these indigenous EOs are cultivated in the provinces of KwaZulu-Natal, the Eastern Cape, the Western Cape, Mpumalanga, and Limpopo. These crops are also commonly cultivated at high altitudes in the Free State and Gauteng provinces. Various government agencies and organizations (The Eastern Cape Development Corporation (ECDC), the national Department of Science and Technology (DST), and the Council for Scientific and Industrial Research (CSIR)) in the Eastern Cape are working together to develop the technology and knowledge needed to implement essential oil-bearing plants for commercial production successfully [50]. They are also exploring ways to help farmers extract the oils from their harvested crops, thus contributing to the sustainability of these oils. This, in turn, promotes economic growth by empowering rural people and reducing their dependence on the government.

**Table 1:** Advantages and disadvantages of different methods of extracting essential oils.

Extraction Method	Principle of Operation	Temperatures	Extraction Time	Yield	Selectivity for Bioactive Compounds	Industrial Scalability	Advantages	Disadvantages	References
Hydrodistillation	Plant material is heated in water to release volatile oils through vaporization and condensation.	100 °C	2–6 h	Moderate	Moderate	High	Simple setup; low operational cost; widely standardized for research	Prolonged extraction, heat-sensitive compounds will be subjected to thermal degradation	[46,47]
Cold pressing	Plant material is mechanically pressed without applying heat to release oils.	Ambient	Minutes to 1 h	Moderate	Low–Moderate	High (for citrus industry)	Preserves natural aroma profile; no thermal degradation	Oil may hold non-volatile residues, restricted mainly to citrus peels	[51]
Steam Distillation	Volatile compounds are vaporized by steam that passes through and then condensed.	100–120 °C	1–4 h	Moderate–High	Moderate	Very high	Widely used industrial method; produces relatively pure essential oils	Not suited for compounds with low heat tolerance, excessive power usage	[47,49]
Microwave-Assisted Extraction (MAE)	Rapid heat is caused by microwave radiation, resulting in bursting of plant cells and release of essential oils.	50–120 °C	10–30 min	High	High	Moderate	Rapid extraction; energy efficient; improved yield	Thermal degradation when temp is uncontrolled, equipment cost	[52]
Ultrasound-Assisted Extraction (UAE)	Ultrasonic waves cause cavitation, which disrupts the plant cell walls and improves solvent penetration.	25–60 °C	15–60 min	Moderate–High	High	Moderate	Reduced extraction time; improved mass transfer	Not easily scalable, solvent is required	[53,54]
Supercritical CO_2_ Extraction (SFE)	Bioactive and aromatic compounds are isolated by high-pressure carbon dioxide.	35–60 °C	30–120 min	High	Very high	Moderate	High purity extracts; no solvent residue; tunable selectivity	Equipment is very expensive, high operational pressure	[55,56]
Enzyme-Assisted Extraction (EAE)	The degradation of the cell wall by enzymes releases EOs found in plant tissues.	30–50 °C	1–24 h	High	High	Low-moderate	Environmentally friendly; improves release of intracellular compounds	High-priced enzymes, enzyme stability issues	[57,58]

Among the wild-harvested and commercially cultivated indigenous essential oil plants are *P. graveolens*, *A. afra*, and *Agathosma betulina*. The essential oils of *P. graveolens*, an aromatic plant with diverse therapeutic properties, exhibit antibacterial, antifungal, and antioxidant activities [59]. The plant demonstrates potential antidiabetic effects through α-glucosidase inhibition [60]. Its extracts, particularly the methanolic extract, show high phenolic content and potent antioxidant, anti-tyrosinase, and anti-urease activities. Additionally, they demonstrate photoprotective properties with high sun protection factor values and moderate antimicrobial effects [58]. The studies above highlight the plant’s potential as a source of bioactive compounds for various applications, including diabetes management, skincare, and antimicrobial treatments. The following table (Table 2) summarizes key indigenous essential oils, detailing their botanical sources (including the plant part used), traditional applications, and scientifically validated biological activities. This compilation highlights the ethnopharmacological significance and potential industrial value of these plant-derived volatile compounds.

**Table 2:** A compilation of indigenous essential oils from selected plants and their documented uses in different fields.

Plant Species	Common Name	Family	Plant Part	Major Chemical Constituents	Traditional Uses	Biological Activities	Geographical Distribution	Conservation Status	References
*Agathosma betulina (P.J.Bergius) Pillans*	Buchu	Rutaceae	Leaves	sabinene, p-cymene, β-pinene, α-pinene, α-thujene, myrcene, limonene, linalool, and terpinen-4-ol. Sabinene	Antibiotic protectant, stomach, cleaning of wounds, kidney, and urinary tract diseases	Antimicrobial, anti-inflammatory	Western Cape	Declining	[47,61]
*Artemisia afra*	African wormwood	Asteraceae	Leaves	Rutin, acacetin, thujone, Artemisia ketone, 1.8-cineole, camphor	Coughs, colds, and malaria	Antimicrobial, anti-inflammatory, and antiparasitic	Gauteng, Limpopo, Eastern Cape, KwaZulu-Natal, and Western Cape	Least concern	[62,63]
*Cymbopogon citratus*	Lemon grass	Poaceae	Leaves and flowers	artemisia ketone, linalool, northujane, verbenone, naphthalene, d-cadinene, hedycaryol, and α- eudesmol	anti-rodent, vermifuge, emetic, anti- infective, and anti-plasmodic; treating morning sickness	Anti-inflammatory	Eastern Cape, Gauteng, KwaZulu-Natal, Limpopo, Mpumalanga, North West, Western Cape	Least concern	[64,65]
*Helichrysum odoratissimum*	imphepho (Xhosa and Zulu), kooigoed (Afrikaans), and phefo, towane (Sotho)	Asteraceae	Aerial parts of the plant	β-pinene, limonene, 1,8-cineole, α-humulene and β-caryophyllene	Treat coughs, colds, and fever. Spiritually, it is burned for cleansing and connecting with ancestors.	Antiseptic and anti-inflammatory	Limpopo, Mpumalanga, KwaZulu-Natal, Eastern Cape, Western Cape.	Least Concern	[66,67]
*Leonotis leonurus*	Wild dagga	Lamiaceae	Leaves	limonene, β-ocimene, γ-terpinene, β-caryophyllene, α-humulene, and germacrene D.	Headache, dysmenorrhea, stomach ache, epilepsy, rheumatic pain, spasms, nervous agitation, improvement of memory, hysteria, and depression	Antioxidant, antimicrobial, anticonvulsant, antidiabetic, and anti-inflammatory.	Eastern Cape, KwaZulu-Natal, Limpopo, Mpumalanga, Western Cape	Least concern	[68]
*Lippia javanica*	Lemon bush	Verbenaceae	Leaves, roots, stem, twigs	Myrcene, carvone, piperitenone, Ipsenone, Linalool, Limonene, Ocimenone, p-cymene, Sabinene, Tagetenone	Colds, wounds, insect repellent, bronchitis, skin disorders, and asthma	Anticancer, antidiabetic, antimalarial, antimicrobial, antioxidant	Eastern Cape, Free State, Gauteng, KwaZulu-Natal, Limpopo, Mpumalanga, North West.	Least concern, not threatened	[69,70]
*Pelargonium graveolens*	Rose geranium	Geraniaceae	Leaves	citronellol, geraniol, citronellyl formate, geranyl formate, iso-menthone, linalool, guaiadiene 6,9, and germacrene D	Diarrhea, urinary stones, liver problems, heavy menstrual flows, and is also helpful for detoxification	antiviral, astringent relaxant, analgesic, and anti-inflammatory	Eastern Cape, Limpopo, Mpumalanga, and Western Cape	Least concern.	[71]

### 3.1. Lippia javanica

**Figure 3:**
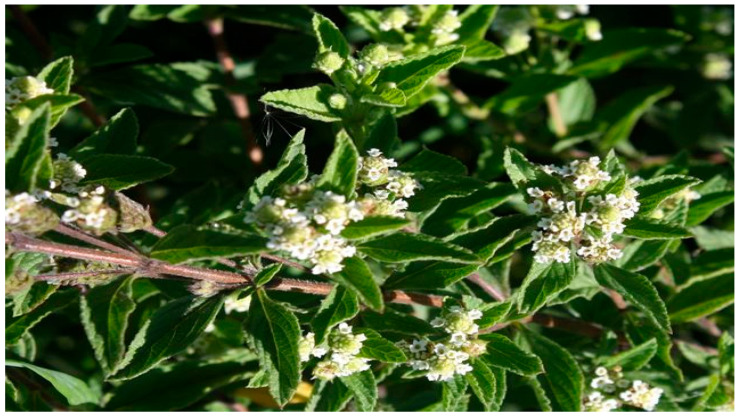
The morphological traits of *Lippia javanica*, characterized by its aromatic leaves and inflorescences [79].

Native to Africa and Asia, *L. javanica* (Figure 3) is an aromatic plant that is traditionally used for culinary and medicinal purposes, and its leaves contain glandular trichomes that secrete secondary metabolites and various elements essential for health [71,72,73,74]. Research has shown that *L. javanica* can help treat respiratory ailments, such as asthma, by suppressing the activation of the T2-mediated immune system and oxidative stress [75]. During the COVID-19 pandemic, there was a rising interest in developing preventive drugs for the disease. Its robust phytochemical composition makes it an ideal candidate for the development of modern herbal remedies to fight infectious diseases [76]. The effects of plant-derived extracts on various health conditions are linked to the modulation of reactive oxygen species (ROS).

Increased ROS generation and oxidative stress have been linked to ageing and neurodegenerative disorders like Parkinson’s and Alzheimer’s diseases, due to the central nervous system’s high vulnerability to ROS-induced damage [77]. An in vitro hepatotoxicity study of *L. javanica* was performed using fluorescent dyes and high-content analysis methods to monitor morphological changes associated with the development of lysosomal dysfunction, myotoxicity, and steatosis [78]. It was observed that the mitochondrial membrane potential was reduced by approximately 10%, but this did not imply a reduction in its content. In addition, the neutral lipid content decreased. The results of the study suggest that the *L. javanica* leaf extract does not pose a hepatotoxic threat at concentrations commonly used in food and topical applications. The lack of sufficient human clinical data and inconsistent study endpoints are some of the key issues that prevent the development of effective therapeutic strategies for liver cancer.

### 3.2. Pelargonium graveolens

*Pelargonium graveolens*, commonly known as rose geranium, has been studied for its phytochemical composition and potential therapeutic applications [80,81,82]. It has been shown that the various extracts exhibited high antioxidant activity, as evidenced by the 2,2′-Azino-bis (3-ethylbenzothiazoline-6-sulfonic acid (ABTS), 2,2-Diphenyl-1-picrylhydrazyl (DPPH), Ferric Reducing Antioxidant Power (FRAP), and Cupric Reducing Antioxidant Capacity (CUPRAC) assays.

**Figure 4:**
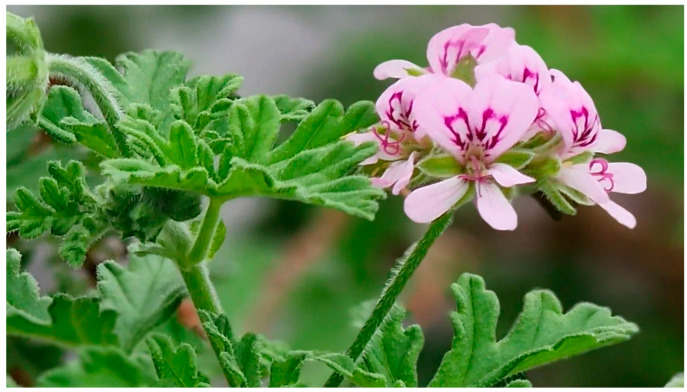
*Pelargonium graveolens* showing the characteristic deeply lobed aromatic leaves and pink flowers typical of this medicinal and essential oil–producing plant [85].

An analysis of the anti-biofilm activity of PGEO using a MALDI-TOF MS system revealed its ability to disrupt the development of *Staphylococcus enterica* on stainless steel and plastic [83]. Evaluations of insecticidal properties revealed that treating the individuals with 100% and 50% PGEO resulted in complete mortality. In addition to its insecticidal and antimicrobial properties, PGEO oil can also be used for therapeutic purposes. Ref. [84] found that PGEO can reduce serum glucose levels and restore antioxidant balance in rats. It was more effective than a drug known as glibenclamide. For the study, the rats were given two 75 mg/kg and 150 mg/kg body-weight doses of *P. graveolens* oil daily for 30 days. Different chemical elements, including liver glycogen, serum glucose, and thiobarbituric acid-reactive substances, were analyzed. The effect of rose geranium (Figure 4) on the hypoglycemic properties of glucose was compared to that of a common anti-diabetic drug.

### 3.3. Agathosma betulina

**Figure 5:**
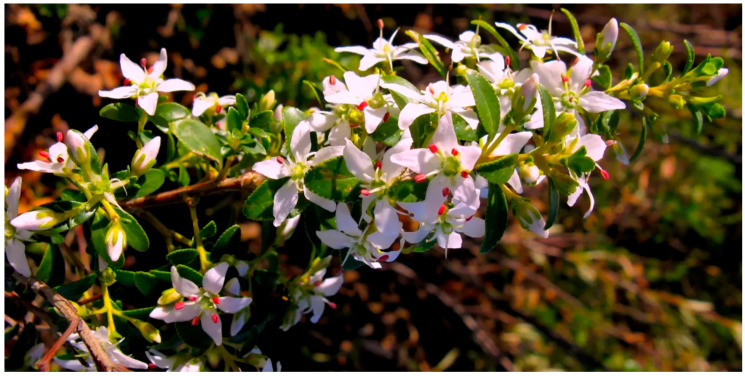
*Agathosma betulina* plant showing the small oil-gland-dotted leaves and star-shaped white to pale pink flowers characteristic of this aromatic medicinal shrub [90].

*Agathosma betulina*, which can be found in South Africa’s Western Cape province, is known for its ability to treat various ailments. It has a long history of being used for its medicinal properties. Despite the prevalence of these traditional uses, scientific validation is still limited. The evidence base supporting these claims is mainly comprised of historical practices and preclinical studies. The commercial potential of the *A. betulina* (Figure 5) plant is enormous, spanning industries such as food flavouring, cosmetics, and pharmaceuticals. Its bioactive compounds, which include pulegone, menthone, and diosphenol, have anti-inflammatory, antimicrobial, and antioxidant properties [86,87]. These properties, including their potential for treating various conditions, are useful in the development of healthcare and biotechnological innovations. This is observed in in vitro and animal studies, which suggest potential benefits for glucose metabolism, weight management, and cardiovascular health [88].

In a study by [89], the authors observed that *A. betulina* exhibits specific growth patterns in its natural habitat, with variations in plant size, leaf morphology, and biomass production. These traits are influenced by environmental factors such as soil composition, altitude, and microclimatic conditions.

### 3.4. Cymbopogon citratus

**Figure 6:**
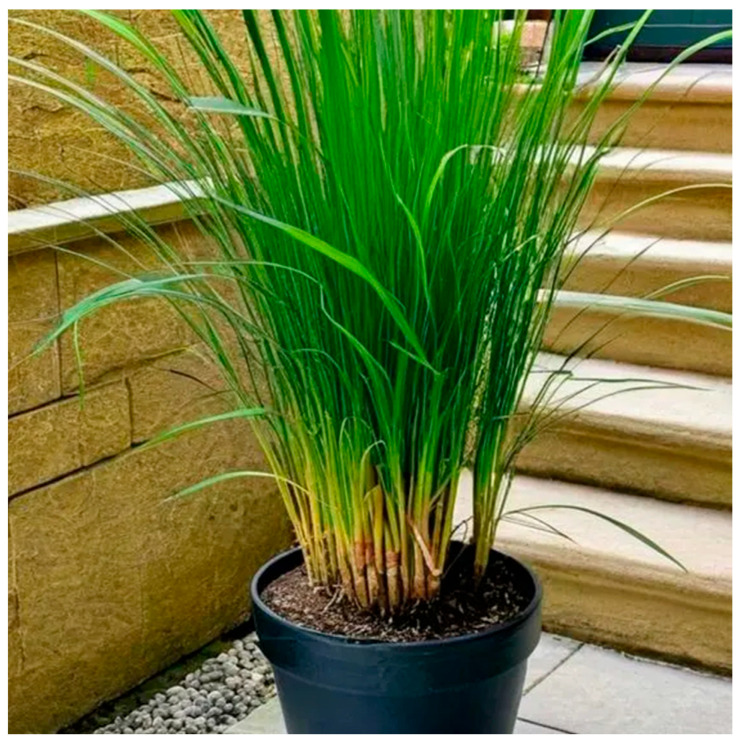
Morphological characteristics of *Cymbopogon citratus*, showing the long, narrow aromatic leaves and tufted growth habit typical of this essential oil–producing grass [98].

Lemongrass is a vital component of the essential oil trade as it is used in a wide range of industries, such as pharmaceuticals, cosmetics, and flavouring. It is usually harvested at around 5 to 6 months old [91]. The plant prefers fertile and well-drained soil types, such as sandy loams, which are ideal for areas with warm subtropical or tropical temperatures. The species can tolerate a wide variety of soil conditions if there is adequate drainage, with its ideal soil pH ranging from 5.5 to 7.5. The *Cymbopogon* genus, including *C. citratus*, is traditionally used for various medicinal purposes, such as antirheumatic, antispasmodic, and analgesic treatments [66]. The essential oils of this plant species are known for their pleasant aromas, which are used in various products, such as beverages and cosmetics (Figure 6). Numerous studies [92,93,94] have been conducted on the properties of these oils, which can repel insects and diseases, and on how they can cure various infections caused by fungi, bacteria, and viruses. Additionally, their use in the fragrance of tobacco and household goods has also been studied [95,96]. Ref. [97] highlighted the potential mechanisms of action of *Cymbopogon* phytochemicals, such as citral’s anti-inflammatory effects and citronellal’s inhibition of the descending pain pathway.

The long-term sustainability of *C. citratus* can be jeopardized by environmental deterioration. Additionally, it is sensitive to defoliation, which can severely affect its sustainability, and its frequent grazing can reduce its population density [91]. While extensive research has been conducted on lemongrass species, including phytochemical analysis and evaluation of biological activities like antioxidant and antibacterial properties, further clinical trials are needed to confirm the safety and efficacy of *Cymbopogon* species in humans [66].

### 3.5. Artemisia afra

**Figure 7:**
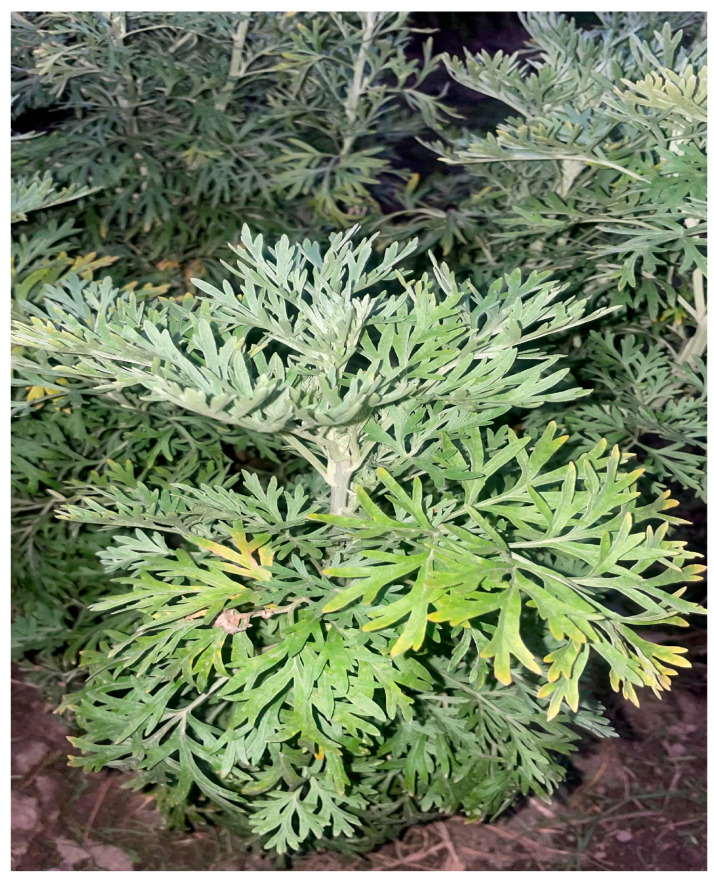
*Artemisia afra*, showing characteristics of its finely divided aromatic leaves and inflorescences (image by Author).

*Artemisia afra* was a widely used remedy during the 1918 influenza pandemic; in 2019, during the outbreak of COVID-19, its use increased. Known for its ability to treat various conditions, such as malaria and coughs, the aromatic plant *A. afra* has gained widespread attention in South Africa. It can also be applied topically to treat measles and hemorrhoids and gargled to treat mouth boils. Fresh leaves can be inserted between the teeth or in the nose to relieve pain and inflammation, and when cooked, the leaves produce vapour that can be inhaled (Figure 7) or ingested to treat respiratory issues [99]. However, there is currently insufficient evidence supporting its effectiveness [100,101]. In vitro and animal studies on *A. afra* have demonstrated only modest biological activity, with recent clinical trials targeting *Schistosoma mansoni* and *Plasmodium falciparum* infections showing promising results, despite some methodological limitations [102].

### 3.6. Leonotis leonurus

*Leonotis leonurus* (L.) R.Br., commonly known as wild dagga, is a perennial shrub indigenous to southern Africa, where it has been traditionally used for its medicinal properties [103]. Traditionally, the flowers and dried leaves of the plant are smoked as a substitute for marijuana in rituals and social settings. In some cultures, the plant is used in cleansing or spiritual practices that aim to enhance one’s connection to nature [104].

**Figure 8:**
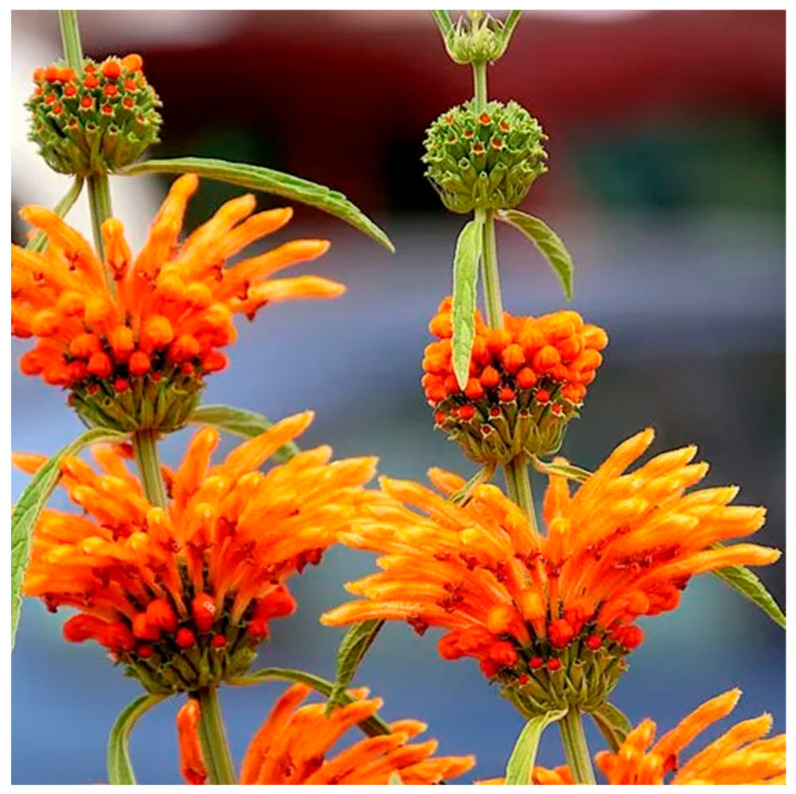
The *L. leonurus* plant, known for its orange verticillaster flower clusters [106].

Phytochemical analyses have identified bioactive constituents, including flavonoids, diterpenoids, and essential oils, which underlie its documented antioxidant and antimicrobial activities (Figure 8). Furthermore, pharmacological investigations have revealed that *L. leonurus* exhibits anticonvulsant, antidiabetic, and anti-inflammatory effects, supporting its ethnomedicinal applications [102].

Despite its widespread traditional use, comprehensive clinical studies are necessary to validate its therapeutic efficacy and safety profile. Notably, while leonurine, a bioactive alkaloid, has been isolated from *Leonurus japonicus*, it is absent in *L. leonurus* and *Leonurus cardiaca*, indicating distinct phytochemical profiles among related species [105].

### 3.7. Helichrysum odoratissimum

**Figure 9:**
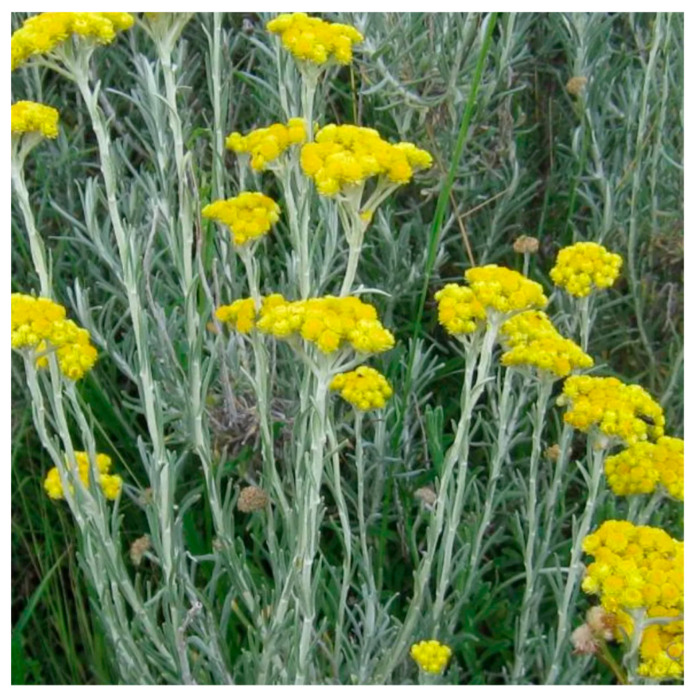
*Helichrysum odoratissimum* with its soft, silvery-grey stem and leaves, with bright yellow floral clusters [111].

The aromatic plant known as *H. odoratissimum* (Figure 9) is native to Southern Africa and has a long history of being used traditionally to chase evil spirits and is believed to bring one closer to their ancestors when burned. A study of the chemical composition of *H. odoratissimum* revealed the presence of various compounds, including 3,4-dicaffeomethylquinic acid and 5,5-dicaffeomethylquinic acid [107]. In a study by [108], it was observed that, relative to corticosterone-treated controls, mice receiving the dicaffeoylquinic acids isomers (3,4- and 3,5) exhibited improved memory performance, reduced production of reactive oxygen species, and lower scores for depressive behaviours. Thus, the high volatility of this EO limits its therapeutic potential. Nanoencapsulation can improve the stability and bioavailability of components in *H. odoratissimum* [109].

Ref. [110] analyzed the nutritional and antioxidant composition of the leaves and stems of *H. odoratissimum*. It was discovered that the leaves had higher protein and crude fat content than the stems. The stems also had a higher neutral detergent fibre content. It was concluded that the tolerable amount of phytate found in both the stem and leaf could be used as a safety factor when it comes to consuming the plant as medicine and food. While studies such as those above have shown the nutritional and bioactive properties of *H. odoratissimum,* the exact therapeutic impact of this plant remains unclear. Most of the evidence supporting its use is derived from animal models; its efficacy in humans is not yet clear.

The lack of clinical trials has limited the ability to confirm the safety and pharmacological properties of this plant. This means that it is important that the clinical studies are conducted to establish the necessary conditions for the safe and effective use of this plant.

## 4. Nanoencapsulation Applications

**Figure 10:**
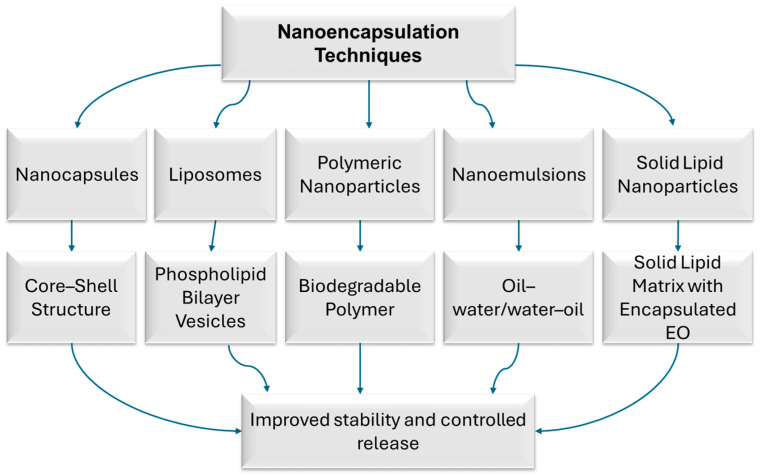
Different nanocarriers loaded with essential oil (created by author).

There are different types of nanoencapsulation techniques, such as those illustrated in Figure 10, in which active compounds can be enclosed within a protective framework, resulting in their stability and restricting their release. These differ by structural arrangement, material, and application [32]. For example, a nano-coacervation involves separating a liquid from a solid in an aqueous solution. This occurs by the interaction of biopolymers, which creates a coacervate layer that holds other materials together [112]. Nanoemulsion is a single-phase, stable, isotropic dispersion consisting of an emulsified oil phase, water, and amphiphilic molecules [113]. The FDA has approved the use of chitosan as a food ingredient. It is a polysaccharide that consists of N-acetyl D-glucosamine units and glucosamine. Due to its affordability and advantages, such as its mucoadhesive and anti-fungal properties, it has become an ideal choice for the delivery of EOs [114]. The physicochemical characteristics of nanocarriers, such as their morphology and pore structure, can significantly affect their loading efficiency and the release kinetics of compounds. Ref. [115] evaluated the performance of nanocarriers by comparing their encapsulation efficiency with two different types of compounds: thymol for volatile compounds and curcumin for non-volatile compounds. After loading, the carriers were vacuum-dried to remove any adsorbed compounds. The effects of the compounds released by the nanocarriers on *Staphylococcus aureus* were studied. The growth rates of bacteria and control groups were also compared with those of the nanocarriers in water. A time-to-detection method was then used to analyze the effects of the compounds on the bacteria. It revealed that the volatilized thymol had a strong antimicrobial effect. A reduction in bacterial count was observed in the presence of 100 µg/mL loaded thymol (0.3 mg/mL SP-A loaded with 34 wt% thymol), and an almost total inhibition of bacteria was attained at a concentration of 300 µg/mL loaded thymol.

The comparison of organic nanocarriers has also been studied. The structural integrity of polymers when used in nanoencapsulation is what makes them ideal for the isolation of sensitive bioactive substances, such as vitamins, essential oils, and drugs. These materials can be distinguished from synthetic polymers due to their structural properties [116]. In a study, Ref. [117] indicates that selecting suitable polymers for encapsulation can assist in determining the stability and viability of functional food systems. Natural polymers like starch, pectin, and alginate can enhance the survival of certain types of bacteria. Additionally, the use of lipid-based materials for the encapsulation of drugs provides a way to protect the drugs from environmental stressors. Another study [118], in agreement with these findings, highlights the various types of polymers that are utilized as wall materials for the encapsulation of drugs in nano-capsules, emphasizing their crucial role in determining the stability and efficiency of the process. Biocompatible polysaccharides and proteins, as well as synthetic polymers with tunable release capabilities, are commonly used. The study highlights the various properties of these materials, such as their ability to form films and their solubility, which influence the performance of the process.

Although the advantages of incorporating polymers into functional food systems are well known, there is no single system that can offer optimal performance in terms of its stability and effectiveness. This paper aims to provide a comprehensive analysis of the various factors that affect the stability and effectiveness of these systems.

Ref. [119] compared the use of solid lipid NPs and chitosan NPs (as carriers of tea tree oil (TTO) against *Staphylococcus aureus* and *Pseudomonas aeruginosa*. It was observed that the encapsulation efficiency of TTO–chitosan NPs was 88.3%, which is higher than that of TTO–solid lipid NPs. The former also exhibited the lowest inhibitory concentrations against both *S. aureus* and *P. aeruginosa* at 35 and 45 ng/mL, respectively. This may be attributed to the size of both these NPs, as chitosan NPs were observed to be 144 nm, while solid lipid NPs were observed to be 477 nm. Moreover, chitosan has antimicrobial properties due to its positive amino groups, while solid lipid NPs are inert and rely on the encapsulation of compounds for their antimicrobial effect. Another study [120] found that the use of nano-liposomes coated with chitosan resulted in better encapsulation of caffeine than nano-liposomes alone. Amongst many wall materials that can be employed in nanoencapsulation are maltodextrin and gum arabic, with their ability to form stable frameworks making them ideal for protecting volatile compounds. Ref. [121] used green ultrasound extraction to extract the bioactive compounds from *Cymbopogon* species and encapsulated them in gum arabic and maltodextrin. Different ultrasound treatments were evaluated for their effects on various parameters, such as the chemical composition, yield, and antimicrobial and antioxidant activities. Treatment with an amplitude of 60% for 60 min was the most beneficial, with a high oil yield and an optimal IC50 value. Gum arabic had the lowest particle size and the best zeta potential in relation to nanoencapsulating. The nanoencapsulated coating successfully extended the shelf life of strawberries.

The various techniques for preparing liposomes, such as solvent injection, thin-film hydration, and reverse-phase evaporation, are discussed [122]. The study highlights their effects on the size, stability, and lamellarity, and it is indicated that the preparation and formulation parameters must be carefully controlled to optimize the characteristics of liposomes for improved drug delivery or controlled release in certain biomedical applications.

South African indigenous EOs are under-researched; however, global studies on related *Lippia* species offer a starting point for comparative insights. Table 3 presents examples of articles reviewed for medicine, food, and agricultural applications of nanoencapsulated indigenous essential oils. Ref. [123] developed a chitosan–caffeic acid (CS-CA) nanogel to encapsulate *Lippia origanoides* essential oil, which resulted in an encapsulation efficiency of 45 ± 2%. In addition, encapsulating the essential oil in the CS-CA nanogel significantly enhanced its antioxidant activity compared to the pure EO. In 2008, Ref. [124] tested a spray-drying process that involved the encapsulation of a small amount of *Lippia sidoides* oil using gum arabic and maltodextrin. The best encapsulation efficiency for the *L*. *sidoides* EO was achieved with a 50% solid content, 100% gum arabic as the carrier, and a 4:1 carrier-to-essential-oil ratio. The results of the study revealed that the oil’s microparticles exhibited antifungal properties.

### 4.1. Medicine

Around 4.2 million people in South Africa are suffering from diabetes, with the most common type being Type 2, which accounts for 90% of all cases. In addition to being a significant public health issue, it is also a leading cause of death in the country [140,141]. *Leonotis leonurus* nanostructured lipid carriers (NLCs) were successfully produced using high-pressure homogenisation (800 bar) with a particle size of 220 nm and a PdI of 0.08. The formulation remained stable under the tested temperatures (4, 25, and 40 °C). When tested on Chang liver and INS-1 cells, the NLCs (1 µg/mL) enhanced glucose uptake and increased insulin release under hyperglycaemic conditions compared to control and non-formulated extract. Overall, NLC encapsulation improved glucose uptake and insulin sensitivity in vitro [133]. It was concluded that encapsulating the *L. leonurus* extract into nanoparticles did not reduce its activity or cause toxicity, and that it retained its potential to improve glucose regulation. This can be attributed to the monoterpenes and sesquiterpenes it contains. The use of nanoencapsulated EOs in disease regulation has been investigated [142,143].

The findings from a study by [19] support the notion that encapsulating EOs could help protect them from the adverse effects of Alzheimer’s disease treatment. In this study, prepared chitosan microparticles (CMs) were loaded with three EOs—*Cymbopogon flexuosus*, *Pelargonium* X *×* ssp, and *Copaifera officinalis*—by spray-drying. Physicochemical and biological characterization was performed. The results showed that both free EOs and chitosan microparticles inhibited acetylcholinesterase (AChE). The reported inhibitory activity of IC_50_ values was in the ~11.9–28.2 µg·mL^−1^ range. Additionally, *Perlagonium* X spp and *C. officinalis* showed higher toxicity to *Artemia salina* and also stronger AChE inhibition than *C. flexuosus*, suggesting greater biological potency.

Drug dosage and how a drug works to block pain signals, induce relief, and enhance the patient’s well-being are the basic concepts of medicine. Ref. [138] investigated the formulated *P. graveolens* EO Nanoemulsion (PGEO-NE). The stabilizer of choice was Tween 80due to its ability to improve the oral absorption of a substance, which is achieved by elevating its solubility and therefore increasing its effectiveness. For nociception tests, prior to the formalin injection, the mice were given morphine, PGEO-NE, or diclofenac salt. They were then placed in an observation tank where they were observed licking or biting their paw after being given the injection. The duration of these actions suggested that they might be nociceptive during the early and late inflammatory phases. The peripheral anti-inflammatory property of PGEO-NE was assessed by measuring the formalin-induced paw edema.

The studies [136,138] revealed a strong correlation between the biological outcomes and the physicochemical properties of the nanocarrier. The characterization and formulation of the nanoemulsion revealed that it has enhanced stability and the ability to deliver lipophilic terpenoids (geraniol and citronellol). The properties of the nanoemulsion directly support the findings of the in vitro study, where it exhibited anti-inflammatory and anti-nociceptive effects. The reduction in the size of the nanoemulsion’s droplets has a beneficial effect on its interactions with biological membranes, which possibly resulted in increased cellular uptake, tissue penetration, and absorption.

The stability of the nanoemulsion can also be attributed to its ability to protect volatile constituents from degradation. This process can result in prolonged therapeutic activity and improve the effectiveness of various inflammatory mediators. Unfortunately, the study [138] highlighted the issue of the degradation of bioactive compounds at elevated temperatures. The results of the two studies revealed that the therapeutic potential of a nanoemulsion depends on the stability of its formulation and storage conditions. Although the studies provide the necessary information to improve the development of new drugs, they also reveal potential constraints that could affect their long-term effectiveness.

*Cymbopogon citratus* was identified as the most effective plant for its antibacterial properties against Gram-positive and Gram-negative bacteria. AgNPs from *C. citratus* exhibited significant antibacterial activity, with an MIC of 25 μg/mL against all tested bacterial species. Higher concentrations of 150 g/mL AgNPs had lethal effects on cells, indicating potential cytotoxicity [128].

Lemongrass oil NPs (LONPs) were effective against *Candida albicans* and *Streptococcus mutans*, with MIC and MBC concentrations of 25 and 100%, respectively. LONPs treatment significantly affected the hardness of acrylic resin at 100% and 50% concentrations, but did not alter its mechanical properties overall. The study concluded that LONPs treatment can decrease microbial growth without compromising the hardness of acrylic resin [125].

### 4.2. Food

Shelf-life extension is an essential factor in improving the quality and safety of food. It can help prevent food waste, improve supply chain efficiency, and boost profit margins. In addition, it can help address global food security issues and achieve sustainable development [144]. Ref. [131] investigated the effects of an oil-in-water emulsion made of PEO and alginate on the lipid oxidation in coconut oil and lard. The measured parameters were 2,2-diphenyl-1-picrylhydrazyl (DPPH) scavenging activity, peroxide value (PV), free fatty acids (FFA), thiobarbituric acid reactive substances (TBARS), colour, and odour acceptability. The results showed that porosity in alginate beads could entrap lipid oxidation products, including volatile compounds and water vapour. Thus, the beads containing the EOs could be used to extend the shelf life of coconut and lard.

By comparing the effect, or rather the impact, of the nanocarrier in both studies [131] and [134] based on application, it can be seen that both studies claim to use nanocarriers to improve the bioactivity of compounds derived from plant oils; the biological outcomes of these systems depend on their composition, functional role, and nature. The fundamental difference between the two systems is the nanocarrier’s active versus passive roles. In the case of geranium oil [131], the latter is mainly passive, resulting in improved bioactivity by virtue of its stability, solubility, and release characteristics. The chitosan-based system [134], on the other hand, is an active nanocarrier that directly participates in the neutralization and antimicrobial action of toxins. It leads to a more complicated mode of action that involves membrane disruption, antioxidant activities, and toxin adsorption. Additionally, in the context of the inhibition of mycotoxins, the nanocarrier can play a dual role by binding to and adsorbing the toxins through electrostatic and hydrogen bonding. It is therefore able to deliver plant-derived antioxidants that can mitigate the effects of oxidative stress caused by toxins.

Nanoemulsions are prepared by using widely recognized emulsifiers, surfactants, and oils. These are generally safer than other nanocarriers, making them suitable for food applications. A recent study [145] revealed that nanoemulsions with 1%, 10%, and 15% concentrations of *C. citratus* oil with uniform spherical structures showed anti-bacterial properties against *Bacillus cereus* and *Staphylococcus aureus*. Additionally, cell viability exhibited a high percentage of cell growth at 2 g/mL.

The incorporation of indigenous EOs into nanosystems can alter their absorption and metabolism in the body. This can result in novel food categories, which can result in lengthy regulatory processes and costly testing. For academic or small-scale projects, this can significantly increase the barrier to entry compared to other cosmetic or aroma products. This may explain why there is limited research on nanoencapsulation of EOs in food.

### 4.3. Agriculture

Agriculture is the backbone of South Africa’s economy, and with climate change, pathogens are becoming resistant to known pesticides and herbicides. A study [132] was conducted where the extract of *H. odoratissimum* was utilized to create zinc oxide NPs. These NPs were designed to address environmental sustainability and food security issues. The research revealed that *H. odoratissimum* serves as an oxygen source and helps reduce particle size. The use of nano-priming technology, as in this study, has revolutionized the field of seed priming, enabling plants to withstand various stresses and improve yields and seed development.

When plants experience vulnerability, such as drought stress and moist storage facilities, fungi such as *Aspergillus flavus* and *Aspergillus parasiticus* develop. The accumulation of these fungi leads to the production of mycotoxins, such as Aflatoxins. A study conducted by [135] on the use of nanoencapsulated *P. graveolens* oil (Ne-PGEO) revealed that the oil can prevent the development of aflatoxin B1 in maize. This was achieved by targeting multiple factors, including the cell membrane’s ergosterol content and cellular ion leakage. The study revealed that the use of Ne-PGEO and free PGEO inhibited the growth and AFB1 production of the *A. flavus* toxigenic strain in a dose-dependent manner. Ne-PGEO significantly decreased the production of AFB1 at a rate of 1.00 μLmL^−1^ as compared to the crude PGEO of 1.25 μLmL^−1^.

In addition to fungal infections, plants are affected by pests such as the rice weevil, which compete for food sources. The rice weevil, known as *Sitophilus oryzae* L., is a widespread, damaging storage pest of cereals such as rice, wheat, barley, and maize. It can cause both quantitative and qualitative losses, as well as seed viability and nutritional deficiencies [146,147]. A study [133] was conducted to improve the stability and utilization of the EO of nano-formulated *P. graveolens* against *S. oryzae* L. The insects were exposed to grain containing different concentrations of *P. graveolens* oil and its nano-formulation. The results indicated that the *P. graveolens* nano-emulsion had the most toxic LC50 value against adults of *S. oryzae*, with an LC50 value of 2.29 ppm/cm^2^ after 72 h. Compared with *P. graveolens* free EO, it has the lowest toxicity with an LC50 value of 67.662 ppm/cm^2^.

### 4.4. Antimicrobial Resistance

Emerging resistance to the antimalarial drug has prompted researchers to find new, innovative ways to combat this resistance. Studies [148,149] have suggested that *A. afra* retained its anti-plasmodial activity despite minimal artemisinin levels. A study by [125] found that encapsulating essential oils in liposomes improved the antimicrobial efficacy of *A. afra, Eucalyptus globulus,* and *Melaleuca alternifolia* compared to the non-encapsulated oils, except for the *A. afra* essential oil. The encapsulated formulations of *E. globulus* and *M. alternifolia* essential oils showed synergistic antimicrobial interactions. Moreover, the polymer coating did not enhance biocidal activity, but it was important for improving the stability and shelf life of the EO formulations. Using plant extracts of *H. odoratissimum*, stabilized gold NPs (AuNPs) were able to effectively fight bacterial biofilms, especially those formed by *Cutibacterium* (formerly known as *Propionibacterium*) acnes. The stabilized particles inhibited *Cutibacterium* acnes cell adhesion and exhibited greater efficacy [150].

A study [139] combining the EO of geranium with solid lipid NPs was developed to control the larvae of *Phthorimaea opoculella* both in vivo and in vitro. The study revealed that solid lipids NPs enhanced larvicidal efficacy compared to bulk oil. It is worth noting that encapsulation can sometimes reduce free oil bioactivity due to altered release kinetics. For instance, the loading process can change the chemical structure of the oil, which can affect its release rate and interaction with the tissues and cells of the target site. Moreover, the carrier system used to transport the oil may dilute its concentration, limiting the amount available at the targeted site. For example, Ref. [131] found that, compared with geranium oil (GO), nanocapsules containing GO did not prevent the development of Candida albicans’ virulence factor.

Biosynthesized NPs showed enhanced antibacterial activity against multidrug-resistant bacteria. Several studies [126,127] addressed the challenge of emerging resistance to antibiotics by developing innovative drug delivery systems. The method is described as a “green approach”, indicating an environmentally friendly and sustainable technique. In one study, the aqueous extract of *C. citratus* was used to synthesize silver NPs (AgNPs) when silver nitrate was used as a precursor, and the formation of AgNPs was confirmed through UV spectral analysis. In another study, levofloxacin-loaded copper oxide NPs using *C. citratus* were employed.

Although the use of nanoencapsulation has been widely touted as a beneficial technique for enhancing essential oils’ bioactivity and stability, a few [151,152,153] studies have produced conflicting results. While it can improve the efficacy of antimicrobial agents, certain processing methods can degrade key compounds, which can result in decreased antimicrobial and antioxidant activity. Additionally, discrepancies in the results of in vitro and in vivo studies regarding the properties of nanoencapsulating systems are a result of the impact of the complex matrices on them. This implies that the nanoencapsulation process is mainly dependent on the oil composition, the delivery system’s physicochemical properties, and the encapsulation technique.

## 5. Mechanism of Action

The efficacy of EOs as antimicrobial and antifungal agents can be attributed to various factors, such as low molecular weight and high lipophilicity, which allow penetration of a bacterial cell. In addition, the composition and concentration of chemical constituents of the EO are the most important factors that affect their effectiveness. One of the advantages of utilizing nanocarriers in EOs is their ability to enhance the efficacy of these substances at lower concentrations [154].

The first encounter occurs through hydrophobic and electrostatic interactions, where nanoencapsulated EOs enhance their adhesion to negatively charged microbial surfaces.

The small size of these components allows them to penetrate biological membranes and extracellular polymeric substances in a biofilm. At the interface between the membranes and the compounds, activation of the degradation or diffusion mechanisms can be controlled. This is where the release of bioactive compounds of the EOs occurs [155].

When bioactive compounds are released into the lipid bilayer, they disrupt the phospholipid packing process and increase lipid mobility. When this occurs, it can result in ion leakage, a decrease in adenosine triphosphate (ATP) synthesis, and protein inactivation. Their intracellular effects can trigger various reactions, such as protein denaturation, protein oxidation, and interference with the replication of nucleic acids [156].

Additionally, mitochondrial dysfunction and the disruption of the electron transport chain can lead to the accumulation of reactive oxygen species. These can cause various reactions, such as DNA damage, apoptosis, and oxidative stress. In nanoencapsulations of Eos, the release can be continuous and precise [157]. A polymeric nanocapsule, for example, can be made with an oleic core that can be used to encapsulate lipophilic substances. It can also be enabled with a polymeric shell that can regulate the release of the drug when needed [158].

A study conducted by [159] to analyze the antifungal properties of chitosan *Pimenta dioica* essential oil (CNPDEO) and without the chitosan (PDEO) against *A. flavus* measured the cellular constituents’ release and the dysfunction of its mitochondria membrane potential. In this study, it was concluded that the latter exhibited a stronger efficacy against the cellular ergosterol synthesis at lower concentrations. Another study [160] evaluated the bacterial proteins and nucleic acid leakage of lemongrass essential oil (LGO) and LGO encapsulated in zein–sodium caseinate nanoparticles (Z-NaCAS NPs). The potential effects of LGO and LGO-loaded Z-NaCas NPs on bacterial cells were evaluated. It was concluded that the absorbance of the cellular components of the treated bacterial cells was 260 and 280 nm higher than that of the untreated cells. The optical densities (OD) were 260 and 280 nm for the maximum peaks of the protein and nucleic acid absorption, which indicated how the tested agent affects the integrity of the bacterial cell membrane. High values of protein and nucleic acid absorption can be used to identify the leakage of proteins and nucleic acids from the bacterial cells. The results of the study revealed that after treating bacterial cells with 1 MIC of unencapsulated non-organic LGO, the cells’ OD260 nm increased by 10.5- and 10.9-fold, respectively. Compared to the untreated cells, the OD260 nm of the nanoencapsulated cells exhibited a significant increase of 9.2-, 12.9-, 8.1-, and 11.6-fold.

The results also revealed that the two compounds decreased the energy-coupling potential of the mitochondria. This phenomenon can be attributed to the changes in the proton concentration in the transport chain. The energy-coupling potential of the mitochondria is regarded as one of the most important biochemical characteristics [161].

The organ’s absorption mechanisms are mainly composed of lymphatic, paracellular, and transmembrane transport. Compared to standard oral formulations, the routes for nano-formulations are significantly more complicated. Besides the paracellular and passive diffusion pathways, nanocarriers can also enter the small intestine through the endocytosis process. They can also access the lymphatic system through the phagocytosis pathway, which is triggered by the M cells in the Peyer’s patches [162].

Ref. [163] developed 2-Monoacylglycerol mimetic liposomes (SER-LPS) by covalently bonding serinol on the surface of the liposomes. The model drug, dihydroartemisinin (DHA), was chosen due to its disadvantages, such as its poor solubility. The exocytosis and endocytosis processes were studied in Caco-2 cells and the monolayers of Caco-2. In addition, the capacity of the intestine’s lymphatic transport was assessed through in vitro and ex vivo biodistribution experiments. The effects of oral administration on the accumulation of drugs in the mesenteric lymph nodes were studied. The levels of DHA obtained from the SER-LPs were 10.40-fold and 1.17-fold higher than those obtained from free DHA or unmodified liposomes. In a similar study, Ref. [164] designed and developed nanoliposomes that can be used to address the challenges of oral administration. They were equipped with a DSC-modified prodrug and a disulfide bond, which can simultaneously deplete the glutathione (GSH) and trigger lethal oxidative stress. This combination of drugs can enhance the anti-malarial effect. In vitro, the polydopamine-modified nanoliposomes (cRPNLs) exhibited a pH-responsive and GSH-dependent release. They were also able to target Peyer’s patches with minimal liver distribution. In vivo, they exhibited a significant reduction in GSH levels and elevated ROS in erythrocytes. The study revealed that they were more effective than free DHA and nanoliposomes. These findings showed that cRPNLs can be a potent oral drug candidate that combines the effects of both parasite-induced drug release and lymphatic uptake.

### Toxicity of Nanocarriers

The regulatory authorities of Southern Africa are aware of the concept and are applying legislation to various medical products that are related to nanomedicines. However, they have no specific definition of what nanomedicines are and how they can be utilized. They also lack cooperation with other organizations and experts in the development of regulations [165]. A few African countries (Egypt, South Africa, Ghana, Tunisia, and Algeria) are attempting to incorporate nanotechnology in their agricultural sector. This means that the region’s policymakers must prioritize the development of policies and programmes that support the use of nanotechnology in order to ensure that it contributes to the continent’s sustainable development [166].

While the use of nanoencapsulation can improve the stability and delivery of bioactive compounds, it is also important to consider the potential toxic effects of these materials on various biological systems. For instance, in maize, the use of TiO2 NPs resulted in enhanced enzyme activities and seed vigour. On the other hand, the use of ZnO NPs led to an increase in salt tolerance in tomatoes. This is attributed to their small size and high surface charge that can lead to unintended toxicological reactions in the environment [167].

The emission sources of NPs have diversified into the soil and aquatic environments. These materials enter the environment through different pathways, such as the release of NPs during the production of raw materials, the application of NPs, and the disposal of these materials [168]. Additionally, the toxicity of various types of nanocarriers (depicted in Table 4) depends on their properties, such as their particle size, composition, and surface charge. While biodegradable and lipid-based nanocarriers are commonly used in agricultural and pharmaceutical applications due to their low environmental impact and superior biocompatibility, the metal-based ones require careful evaluation because they can induce cellular damage and oxidative stress [169]. It is therefore recommended that, in order to ensure the safety and sustainability of nanotechnology, one must understand these factors and employ suitable design methods. The surface chemistry of these materials determines whether they are toxic or not. In a study conducted by [170], the eight different types of nanocarriers, namely poly(ethylene glycol) polymers, neutral and cationic liposomes, micelles, poly(amindo amine) and poly(propyleneimine) dendrimers, quantum dots, mesoporous silica, and supermagnetic iron oxide (SPIO) nanoparticles, were evaluated and the effects of their charge on the formation of micronsuclei were shown to be a marker of their toxicity. Cationic carriers had the most significant effects, while neutral and negatively charged ones did not. In studies conducted [171,172] in 2020 and 2021, respectively, the authors noted that metal-based and protein-based nanoparticles can cause oxidative stress and infiltrate cell nuclei. On the other hand, Ref. [173] noted that certain types of nanocarriers, such as dendrimers, liposomes, and dendriplexes, have minimal toxicity. Different coating methods were used to evaluate the properties of nanocapsules. In a study [174], it was discovered that chitosan-coated nanocapsules were toxic regardless of their exposure. In determining the viability of a cell depends on its surfactant and polymer composition. Eudragit RL 100 exhibited greater cytotoxicity than PLGA, PCL, or chitosan nanoparticles.

In addition to the damaging effects of oxidative stress, NPs can also trigger an inflammatory response through the release of cytokines, which are produced by certain signalling cascades. In vivo studies have shown that these particles can accumulate in various organs, including the liver, lungs, brain, and spleen, which can lead to various toxic effects [175].

## 6. Challenges and Limitations

### 6.1. Socio-Economic and Production Challenges

To assess the socio-economic and production challenges in the Eastern Cape, the experiences and lessons learned from the Qobo Qobo and Bulungula Incubator initiatives will be examined. One of the most significant barriers to entry for small-scale farmers is the high cost of production in the EOs market. To combat this, the Qobo Qobo project integrates more small-scale farmers into its programme. On the other hand, one of the goals of the Bulungula Incubator is to elevate its community through partnerships with various organizations, including government agencies and the community itself. The cooperative’s primary business is the sale of dried lemongrass, which is used in the production of tea and essential oils. Some of the challenges this initiative has encountered include infrastructure, demand for lemongrass (which has led to part of the space being transformed into crop planting), youth participation, and certification. The cooperative decided to diversify its offerings after noticing that the quality of its dried lemongrass had been compromised.

Another challenge is the oral transmission of indigenous knowledge, which is common. This is an issue as it can threaten its preservation when elders pass away or when the next generation is no longer acquainted with traditional practices. In addition, indigenous groups protect their sacred knowledge by keeping it a secret. This prevents outsiders from misinterpreting or exploiting it. Moreover, both Western science and indigenous knowledge systems (IKS) have their own distinct worldviews. For instance, Western science usually adopts a reductionist, analytical perspective, while IKS is more empirical and integrative.

A way forward from these challenges would be to adopt community-led documentation, such as participatory research, in which knowledge holders and elders create visual, audio, or written records of traditional practices. Therefore, instead of imposing academic methods on the community, use culturally appropriate recording techniques. These can reflect local language, storytelling, and symbolism. Another approach would be to train young individuals in data management, ethnobotany, and documentation to help ensure the preservation of indigenous knowledge.

### 6.2. Scientific Challenges

Scientific studies on most indigenous plants are limited, and the majority of them lack the necessary scientific testing to ensure their safety and efficacy. Additionally, nanotoxicity remains a significant concern. Studies [164,165,166] have indicated that the accumulation of NPs can lead to plant toxicity. This can be caused by the generation of excess reactive oxygen species, which can affect cellular biomolecules. Although NPs can be toxic in plants and humans at any given stage, their toxicity is influenced by various factors. Some of these include their concentration, physical properties, and their ecological ageing. Additionally, the potential risks associated with nanotoxicity to aquatic systems and humans are significant. These include genotoxic and cytotoxic effects in human cells, as well as disruption of microbial communities in soil and water. These concerns highlight the need to conduct comprehensive assessments of the toxicological effects of nanotechnology to establish a safer framework for its use.

Another limitation is that there is a limited amount of research on indigenous plants for nanoencapsulation, which is due to the complexity of optimizing a nanocarrier’s delivery system. One has to consider the various characteristics of a nanocarrier material before choosing it for a particular application. For instance, the type of bioactive compound you want to deliver, the surface groups it interacts with, and the processing parameters are all taken into account to determine the best choice.

While socio-economic and scientific barriers exist, addressing them through interdisciplinary research, policy support, and technological innovation is essential for advancing nanoencapsulation of indigenous EOs.

## 7. Conclusions and Recommendations

It was noted that amongst all the EOs reviewed in this study, *Lippia javanica* is the most studied. This may be due to it being a multifaceted organism that has diverse ecological and biochemical characteristics. Additionally, its scientific significance can be attributed to its convergence of diverse biological activities, ethnobotanical relevance, and phytochemical richness.

From the nanotechnology point of view, despite the promising laboratory studies as described in the literature, research has not yet progressed to human clinical trials for nanoencapsulated indigenous EOs, for example in diabetes management and other diseases. The safety implications and toxicity resulting from nanomaterial accumulation require additional research for proper validation. Although extensive global research has examined the potential of nanoencapsulation of EOs, little is known about the various applications of South African indigenous species. This gap in the research literature highlights the significance of this review, which aims to encourage the exploration of these local resources. The current review’s originality and its contribution to bridging regional and scientific knowledge gaps can help foster sustainability and innovation.

Several indigenous EOs can have unique chemical properties and require customized nanoencapsulation techniques. The interactions between the carrier and oil are essential in determining the stability and effectiveness of nanoencapsulated oils. Varying surface charge, binding affinity, and composition can affect the release kinetics and efficiency of these materials. To optimize the formulation, it is necessary to evaluate these interactions through performance testing and thorough characterization. Further research should examine the safety and effectiveness of nanoencapsulation across various applications and explore novel approaches to improving encapsulation. As more research is conducted, clear guidelines on safe dosage ranges, optimal encapsulation levels, and regulatory standards should be developed.

Additionally, the approach of nanoencapsulation can help small-scale producers by improving the quality and shelf life of the EOs they produce. It can also help them gain access to the larger market and create value-adding opportunities in local economies.

The information provided in this review is valuable as it highlights the combination of nanotechnology and indigenous knowledge, which has the ability to unlock South Africa’s economic potential. This can help foster innovation that is both globally competitive and culturally rooted.

### 7.1. Conclusions

This review demonstrates that indigenous South African essential oils (EOs) possess significant pharmacological, agricultural, and food-preservation potential. Species such as *Lippia javanica*, *Pelargonium graveolens*, *Agathosma betulina*, *Artemisia afra*, *Leonotis leonurus, Cymbopogon citrutas*, and *Helichrysum odoratissimum* contain diverse bioactive compounds (terpenes, phenolics, aldehydes) that exhibit antimicrobial, antioxidant, anti-inflammatory, antidiabetic, and insecticidal activities. These properties position them as promising alternatives to synthetic preservatives, agrochemicals, and certain pharmaceutical agents.

However, the practical application of EOs is constrained by their volatility, poor aqueous solubility, instability under environmental stressors, and strong aroma profiles. Nanoencapsulation technologies, including liposomes, nanoemulsions, solid lipid nanoparticles, and polymeric nanogels, offer effective solutions by enhancing stability, bioavailability, controlled release, and targeted delivery. Evidence from reviewed studies indicates improved antimicrobial efficacy, enhanced glucose regulation activity, extended food shelf life, and superior pest and fungal control when EOs are nanoformulated.

Despite these advances, major gaps remain. Most studies are limited to in vitro or animal models, with insufficient human clinical validation. Nanotoxicity concerns, regulatory uncertainty, limited commercialization pathways, and socio-economic barriers for smallholder farmers restrict large-scale adoption. Overall, nanoencapsulation represents a transformative platform capable of unlocking the full value of indigenous essential oils, provided that scientific validation, regulatory frameworks, and inclusive economic models are strengthened.

Moreover, the assessment of nanotoxicity in food and medical products is carried out according to the recommendations of existing safety guidelines. Various organizations, such as the Economic Co-operation and Development (OECD), provide standard testing protocols. The European Medicines Agency (EMA), European Food Safety Authority (EFSA), and the FDA have also developed their own guidance for the use of nanomaterials. The frameworks that are currently in place provide a comprehensive assessment of the physicochemical properties of nanomaterials and their effects on human health. However, they do not provide a standardized testing method for nano-specific compounds. This issue highlights the need for more effective and harmonized regulations.

### 7.2. Recommendations

To advance the development and application of nanoencapsulated indigenous essential oils (EOs), several strategic interventions are recommended. Stronger scientific validation is required to enhance credibility and regulatory acceptance. Well-designed preclinical and clinical studies should be conducted to confirm safety profiles, optimal dosage ranges, and therapeutic efficacy. Standardized extraction procedures and comprehensive phytochemical profiling must be established to ensure consistency, quality control, and reproducibility across studies. In addition, expanded toxicological assessments are necessary to evaluate potential long-term nanotoxicity effects not only in humans but also in plants, soil ecosystems, and aquatic environments, particularly where agricultural applications are envisaged.

Additionally, clear regulatory and policy frameworks should be developed to guide the safe commercialization of nano-enabled herbal and food products. National guidelines specific to nanoformulated botanical products are needed to address safety testing, labelling, and market authorization requirements. Harmonizing safety and quality standards with international best practices will further enhance export readiness and global competitiveness. Simultaneously, mechanisms to protect indigenous knowledge systems through intellectual property rights and equitable benefit-sharing agreements should be strengthened to ensure that originating communities derive fair socio-economic returns.

Sustainable cultivation and conservation strategies are equally important. For species with declining natural populations, such as *Agathosma betulina* (buchu), cultivation should be prioritized over wild harvesting to prevent resource depletion. Conservation of natural habitats must also be integrated into production strategies, as environmental conditions significantly influence phytochemical composition and oil quality.

To support commercialization and inclusive value chain development, investment in pilot-scale nanoformulation facilities within the province is recommended to reduce reliance on external processing and increase local value addition. Public–private partnerships linking farmers, researchers, and industry stakeholders should be strengthened to accelerate technology transfer and market integration. Capacity-building initiatives are also necessary to equip smallholder farmers with skills in quality control, certification standards, and market access strategies.

Preservation and integration of indigenous knowledge systems remain central to sustainable innovation. Community-led documentation initiatives using culturally appropriate recording methods should be promoted to safeguard traditional knowledge associated with essential oil use. Training programmes in ethnobotany, digital documentation, and EO processing should target youth to facilitate intergenerational knowledge transfer. Furthermore, collaborative research models that respectfully integrate indigenous knowledge with modern scientific methodologies should be encouraged to ensure culturally sensitive and ethically grounded innovation.

Finally, application-specific research should be expanded to maximize sectoral impact. In medicine, future studies should prioritize chronic diseases prevalent in South Africa, including diabetes and antimicrobial resistance. Within food systems, research should focus on the development of active packaging technologies and natural preservative systems that enhance shelf life, reduce post-harvest losses, and contribute to food security.

### 7.3. Future Studies

Future research (represented in Table 5) should prioritize strengthening the scientific, technological, and translational foundations of nanoencapsulated indigenous essential oils. Rigorous preclinical and clinical investigations are needed to establish safety profiles, optimal dosage parameters, pharmacokinetics, and long-term efficacy, particularly for chronic conditions relevant to South Africa, such as antimicrobial resistance and metabolic disorders. Greater emphasis should also be placed on comprehensive toxicological studies to evaluate potential nanotoxicity in humans, food matrices, soil systems, and aquatic environments.

From a technological perspective, research should focus on optimizing nanoencapsulation techniques to improve encapsulation efficiency, scalability, cost-effectiveness, and stability under real processing and storage conditions. Future work should also expand into regulatory science and standardization, including the development of harmonized safety assessment protocols and quality control benchmarks tailored to nano-enabled botanical products. Studies exploring life cycle assessment (LCA) and environmental impact modelling will be critical to ensuring sustainable scale-up.

Finally, interdisciplinary and community-engaged research models should be strengthened to integrate indigenous knowledge systems with advanced nanotechnology. Socio-economic impact assessments, value chain analyses, and market feasibility studies will be essential to ensure that innovation translates into inclusive commercialization pathways that benefit local communities and support rural economic development.

## Figures and Tables

**Table 3:** Exploring the role of indigenous plants in nanoencapsulation procedures and their applications in various scientific and agricultural applications.

Plant Material	Plant Species	Method	Size	Encapsulation Efficiency (%)	Application	Reference
Plant oil	*A. afra*	Polymer-coated liposomes	8.269 ± 0.796 µm	18.7	Medicine	[125]
Crude extract	*C. citratus*	CuO NPs	55 nm	Not reported	Agriculture	[126]
Plant extract	*C. citratus*	Ag NPs	47 nm	Not reported	Medicine	[127]
Plant extract	*C. citratus*	Ag NPs	~35 nm	Not reported	Agriculture	[128]
Plant oil	*C. citratus*	Nanoemulsion	10–100 nm	Not reported	Medicine	[129]
Plant oil	*C. citratus*	Liposome	200 nm	Not reported	Medicine	[130]
Plant oil	*Geranium*	Alginate beads	Not reported	Not reported	Food	[131]
Plant extract	*H. odoratissimum*	ZnO NPs	144.6 ± 2.60 nm	Not reported	Agriculture	[132]
Plant extract	*L. leonurus*	Nanostructured lipid carriers	220 nm	Not reported	Medicine	[133]
Plant extract	*L. leonurus*	Chitosan nanoparticles	31–65 nm	Not reported	Food	[134]
Plant oil	*P. graveolens*	chitosan-cinnamic acid nanogel	Not reported	30.3 ± 1.77–38.4 ± 1.37%	Agriculture	[135]
Plant oil	*P. graveolens*	Nanoemulsion	164 nm	Not reported	Medicine	[136]
Plant oil	*P. graveolens*	Nanoemulsion	30.99 nm	Not reported	Agriculture	[137]
Plant oil	*P. graveolens*	Nanoemulsion	553 nm	Not reported	Medicine	[138]
Plant oil	*P. graveolens*	Solid lipid nanoparticles	50–170 nm	~ 74.93–93.46%	Agriculture	[139]
Plant oil	*Pelargonium ×* ssp	chitosan microparticles	2–3 μm	35.2–58.5%	Medicine	[19]

**Table 4:** Different types of nanocarriers and their specific characteristics.

Nanocarrier	Common Materials	Biotolerance	Major Toxicity Mechanisms	Advantages	Toxicity Concerns	Application	Reference
**Liposomes**	Phospholipids	Remarkably high	Harmless. Can trigger an immunological response in some cases.	Imitates biological membranes, biodegradable	Degradation, Lipid peroxidation	Essential oil encapsulation	[175,176]
**Solid Lipid NPs**	Nonpolar, hydrophobic lipids (Fatty acids, Triglycerides)	High	Surfactants can induce a cyclic cytotoxic reaction, e.g., lipid oxidation.	Stable, controlled release; low toxicity	Nucleation, limited encapsulation ability	Agriculture, pharmaceuticals and cosmetics	[177,178]
**Nanostructured Lipid Carriers (NLCs)**	Combination of solid and liquid lipids	High	Identical to SLNs; however, lower toxicity due to improved lipid structure	Stable, Improved encapsulation	Surfactants can cause irritation at high concentrations	Drug delivery, nutraceuticals	[179,180]
**Polymeric NPs**	Polyethylene glycol, Poly(lactic-co-glycolic acid), alginate, chitosan	Moderate-high (dependent on polymer)	Membrane disruption (cationic polymers)	Tunable properties	Cytotoxicity	Nanoencapsulation of EOs and drug delivery	[181]
**Chitosan NPs**	Chitosan	High	A strong positive charge can disrupt a membrane.	Antimicrobial, biodegradable	Possible cytotoxicity at high concentrations	Antimicrobial preparations and agriculture	[182,183]
**Nanoemulsions**	Oil, water, surfactants	High	Surfactant toxicity	Easy to prepare, efficient dissolution rate for hydrophobic substances	Concentration of surfactant determines stability	EO delivery, food, and cosmetics	[177,184,185]
**Dendrimers**	Poly(amidoamine), Polypropylene Imine (PPI) dendrimers	Moderate	The strong interaccumulation between a nanocarrier and a cell membrane or a cytotoxic surface group can lead to toxicity.	High encapsulation, controlled structure	Surface-dependent toxicity	Drug delivery and gene transfer	[178,179]
**Metallic NPs**	Ag, Au, ZnO	Low-moderate	DNA damage, ROS generation, and mitochondrial dysfunction.	Highly potent	Environmental residue, high cytotoxicity	Antibacterial coatings and sensors	[181]
**Silica NPs**	Mesoporous silica	Moderate	Tissue-level stress response, Oxidative stress.	Porous	Environmental persistent	Catalysts and drug delivery	[185,186]

**Table 5:** Future Research Agenda for Nanoencapsulated Indigenous South African Essential Oils.

Research Priority	Key Research Needs	Potential Impact for Food Systems
Clinical validation	Conduct preclinical and clinical studies to confirm safety, efficacy, and dosage of nanoencapsulated essential oils, particularly for antimicrobial and metabolic health applications	Scientific validation supporting nutraceutical and functional food development.
Toxicology and safety assessment	Evaluate nanotoxicity, long-term safety, and environmental impacts in food matrices, soil ecosystems, and aquatic environments. Develop standardized safety protocols	Safer application of nano-enabled preservatives and increased regulatory acceptance
Phytochemical standardization	Establish standardized extraction methods and perform detailed phytochemical profiling to identify active compounds and chemotypes	Consistent quality and reproducibility of essential oil-based ingredients
Food preservation and packaging	Develop nanoencapsulated EO-based preservatives and antimicrobial active packaging. Assess shelf-life extension and sensory impacts in real food matrices	Reduced food spoilage, improved shelf life, and natural alternatives to synthetic preservatives
Agricultural and Pest Management Applications	Develop nanoformulated essential oil-based biopesticides and evaluate their effectiveness within integrated pest management systems	Sustainable crop protection alternatives to synthetic agrochemicals.
Sustainable Cultivation and Conservation	Develop cultivation protocols for key species and investigate environmental influences on oil yield and chemical composition. Promote domestication of high-value aromatic plants.	Sustainable supply of essential oil resources and conservation of biodiversity
Regulatory and commercialization pathways	Develop regulatory guidelines, quality standards, and value chain models to support commercialization of EO-based nano-products.	Market-ready products, enhanced industry uptake, and rural economic development
Indigenous knowledge integration	Document traditional uses of aromatic plants and integrate ethnobotanical knowledge into scientific innovation frameworks	Ethical innovation and inclusive value chains benefiting local communities

## Data Availability

No new data were created or analyzed in this study. Data sharing is not applicable to this article.
